# Organelle and Cellular Abnormalities Associated with Hippocampal Heterotopia in Neonatal *Doublecortin* Knockout Mice

**DOI:** 10.1371/journal.pone.0072622

**Published:** 2013-09-02

**Authors:** Reham Khalaf-Nazzal, Elodie Bruel-Jungerman, Jean-Paul Rio, Jocelyne Bureau, Theano Irinopoulou, Iffat Sumia, Audrey Roumegous, Elodie Martin, Robert Olaso, Carlos Parras, Carmen Cifuentes-Diaz, Fiona Francis

**Affiliations:** 1 INSERM UMRS 839, Paris, France; 2 Université Pierre et Marie Curie, Paris, France; 3 Institut du Fer à Moulin, Paris, France; 4 Centre de Recherche de l'Institut du Cerveau et de la Moelle épinière, Paris, France; 5 INSERM UMRS 975, Paris, France; 6 CNRS UMR 7225, Paris, France; 7 Plateforme de Transcriptomique, Laboratoire de Recherche Translationnelle, CEA/DSV/IG-Centre National de Génotypage, Evry, France; Hospital Nacional de Parapléjicos – SESCAM, Spain

## Abstract

Heterotopic or aberrantly positioned cortical neurons are associated with epilepsy and intellectual disability. Various mouse models exist with forms of heterotopia, but the composition and state of cells developing in heterotopic bands has been little studied. *Dcx* knockout (KO) mice show hippocampal CA3 pyramidal cell lamination abnormalities, appearing from the age of E17.5, and mice suffer from spontaneous epilepsy. The *Dcx* KO CA3 region is organized in two distinct pyramidal cell layers, resembling a heterotopic situation, and exhibits hyperexcitability. Here, we characterized the abnormally organized cells in postnatal mouse brains. Electron microscopy confirmed that the *Dcx* KO CA3 layers at postnatal day (P) 0 are distinct and separated by an intermediate layer devoid of neuronal somata. We found that organization and cytoplasm content of pyramidal neurons in each layer were altered compared to wild type (WT) cells. Less regular nuclei and differences in mitochondria and Golgi apparatuses were identified. Each *Dcx* KO CA3 layer at P0 contained pyramidal neurons but also other closely apposed cells, displaying different morphologies. Quantitative PCR and immunodetections revealed increased numbers of oligodendrocyte precursor cells (OPCs) and interneurons in close proximity to *Dcx* KO pyramidal cells. Immunohistochemistry experiments also showed that caspase-3 dependent cell death was increased in the CA1 and CA3 regions of *Dcx* KO hippocampi at P2. Thus, unsuspected ultrastructural abnormalities and cellular heterogeneity may lead to abnormal neuronal function and survival in this model, which together may contribute to the development of hyperexcitability.

## Introduction

*Doublecortin* (*DCX*) is mutated in subcortical band heterotopia (SBH), a severe human cortical malformation characterized by cortical neuronal somata present in the white matter. Heterotopic neurons [1] are associated with epilepsy, and there is a strong correlation between drug-resistant forms and severe white matter heterotopia [2], [3]. SBH is included in the lissencephaly spectrum [4], which is characterized by a smooth brain surface and a thickened and severely disorganized neocortex, as well as abnormally formed hippocampi [5], [6]. *DCX*, *LIS*1, *reelin* (*RELN*) and *alpha tubulin 1A* (*TUBA1A*) are mutated in these disorders [7]–[11] with *DCX* being the most frequently mutated gene in SBH [12]–[13].

Heterotopic neurons arise during development by a variety of mechanisms [1]. Neurons born close to the ventricles must migrate long distances to reach their final position in the cortical plate [14]. Slowed or arrested migration can therefore lead to abnormal final positioning of neurons in the migratory path [15]. The physiopathological consequences of heterotopia and especially their link with the emergence of epileptiform activities are not well understood. Rare histological and immunohistochemical studies of human heterotopia have shown that they contain both pyramidal cells and interneurons, and DiI tracing studies have revealed connections between heterotopic regions and subcortical/cortical regions [16]. More recent data in rodent models of SBH suggest that not only the heterotopia, but also the overlying cortex function abnormally [17]. However, few studies have been devoted to characterizing the morphological and ultrastructural features of neurons developing in the heterotopic and overlying cortex. This could provide clues to their later abnormal function in the adult.

Mutant mouse lines generated for genes involved in SBH and type 1 lissencephaly in human are consistently associated with heterotopic pyramidal cells in the hippocampus. *Reeler* mice are the most severely affected, showing a grossly disorganized hippocampus and isocortex [15], [18]. *Lis*1 mutant mice have more subtle defects in the isocortex but also exhibit severe hippocampal lamination defects, consisting of fragmented CA1 and CA3 pyramidal cell layers, and this is associated with epilepsy [19]–[21]. *Tuba1a* mutant mice show a similar hippocampal phenotype [11], whilst *Dcx* KO mice present a pyramidal cell disorganization largely restricted to the CA3 region [6], [22]. Interneuron migration abnormalities have been shown to accompany the hippocampal lamination defects in *Lis*1 and *Dcx* mutants [23], [24].

During embryonic development of the WT hippocampus, neurons migrate from the ventricular zone (VZ) of the medial wall across an intermediate zone (IZ, the future *stratum oriens*) to reach the hippocampal plate [25]. In the *Dcx* KO, as well as a correctly forming pyramidal cell layer, an abnormal high density of cells is observed in the IZ during this developmental period [6]. In the adult, *Dcx* KO CA3 pyramidal cells are arranged in two distinct layers, compared to a single layer in WT. Furthermore, mice suffer from spontaneous epilepsy and the CA3 region shows enhanced excitability *in vitro* [26], [27]. Since hippocampal organization is well characterized in WT, *Dcx* KO mice provide an excellent model to further study specific features of developing heterotopic cells, and the generation of hyperexcitability.

[TUGHTER]In WT, interneurons and oligodendrocyte precursor cells (OPCs) originate in the ventral telencephalon during embryogenesis, and migrate long distances to reach medial parts of the cortex, with interneurons reaching the CA3 region by E16 [28]–[31]. In late embryonic stages and postnatally, interneurons and OPCs move within the hippocampus to their final positions [28], [32]. Dentate gyrus granule cell production within the hippocampus temporally matches the other cell types [33], with numerous cells produced from E16 onwards [34], migrating in a tangential subpial stream, to reach the dentate gyrus region [35], where production continues postnatally [28]. During development, cell death is also a physiological phenomenon with peaks of apoptosis observed in the rodent hippocampus in early postnatal stages [36]–[38].

In this study, we set out to characterize the *Dcx* KO CA3 region postnatally, performing an ultrastructural and cellular study to query the nature of the lamination defect. Few ultrastructural analyses examining the content and aspect of heterotopic cells have been reported previously [39], [40]. Organelle modifications were identified in *Dcx* KO neuronal cells including evidence of damaged mitochondria, and more dilated, circular and vesicular Golgi apparatus forms compared to WT. In addition, the postnatal *Dcx* KO pyramidal layers showed a heterogeneous cell composition, exhibiting some interspersed OPCs and other cell types. Caspase-3 dependent cell death was also increased approximately two-fold. These combined data reveal abnormalities in the postnatal *Dcx* KO hippocampus which have not previously been described and which may provide clues to the abnormal functioning of *Dcx* KO pyramidal neurons in the adult.

## Materials and Methods

### Ethics statement, mouse breeding, genotyping and tissue preparation

Animals were treated according to the French Agriculture and Forestry Ministry guidelines (decree 87849, establishment number B75-05-22, individual license numbers 75–654 and 75–1151), with protocols submitted to the local Charles Darwin ethical committee for animal experimentation (C2EA-05).

For each experiment, *Dcx* KO and WT mice maintained on the Sv129*Pas* background were analyzed from several litters. Mice were maintained after more than ten generations of backcrosses and genotyped to verify that the *Dcx* gene was inactivated [24]. Mice were generated by crossing heterozygote females with pure Sv129*Pas* males (Charles River, France). For all analyses male hemizygote KO mice were compared with littermate male WT mice. Newborn P0-P4 animals were anesthetized by hypothermia for 3–4 min. Prior to sacrifice, adult mice were deeply anesthetized by intraperitoneal injections of pentobarbital. For *in situ* hybridization and caspase-3 immunostaining, mice (n = 3 WT and KO) were perfused with 4% paraformaldehyde (PFA) in 0.1 M phosphate buffer (PB), and postfixed in the same solution overnight. Following postfixation, brains were cryoprotected in 30% sucrose, frozen in embedding medium and cut using a cryostat at 16–20 µm. To characterize cell heterogeneity, different neural precursor marker antibodies were used. For these experiments, neonatal P0 mice were anesthetized and perfused with 2% PFA in PB, and postfixed in the same solution for 2–4 hours. Following postfixation, brains were cryoprotected in 30% sucrose, frozen in embedding medium and cut using a cryostat at 12 µm. For electron microscopy (EM) experiments, P0 mice (n = 4 WT and KO) were anesthetized and perfused with 4% PFA and 2.5% glutaraldehyde in PB. The brains were removed and placed in fresh fixative overnight at 4°C, rinsed in PB, postfixed in 2% OsO_4_ (in PB), dehydrated in an ascending series of ethanol, and embedded in epoxy resin. For laser capture microdissection, 4–5 KO and WT P0 mice were anesthetized by hypothermia, and brains flash frozen by dropping in isopentane maintained at −35°C for 2 minutes.

### *In situ* hybridization

Specific antisense RNA probes were generated for *Wfs1* (gene ID NM_011716), *Necab2* (gene ID, NM_054095 and [41]), *KA1* (NM_175481) and *Sst* genes (NM_009215). *Wfs*1 oligonucleotide sequences were forward TACGCCAAGGGCATCATT, reverse CACCAGGTAGGGCACCAG; *Necab*2 forward TACCATCGATTCAGACAACACC, reverse AGGTACTGTCTCAGGGAATCCA; *KA*1 forward CAGCGCATGGAGGTGCCCAT, reverse GGCTCGCTGCTGTTGGTGGT, Sst forward ACGCTACCGAAGCCGTC, reverse GGGGCCAGGAGTTAAGGA. Sections were rinsed in phosphate buffered saline (PBS) and treated with proteinase K (10 μg/ml) for 10 min, blocked in PBS-glycine (2 mg/ml), and hybridized at 70°C overnight with digoxygenin (DIG)-labeled probes diluted 1/100 in hybridization buffer (50% deionised formamide, 10% dextran sulphate, 1 mg/ml Yeast RNA, 1× Denhardt's solution, products obtained from Sigma, France). Sections were then sequentially washed in 2× saline sodium citrate (SSC) Tween-20 0.1% at 70°C then in maleate buffer (maleic acid 100 mM, NaCl 150 mM, 0.1% Tween-20, pH 7.5) at room temperature [42]. For immunological detection, sections were treated in 2% blocking reagent (Roche Diagnostics, France, cat. 1096176, 20% sheep serum in maleate buffer) and incubated overnight at 4°C in the same solution containing sheep anti-DIG-alkaline phosphatase-conjugated Fab fragments (Roche) diluted 1/2000. Sections were then washed in maleate buffer, and in NTMT buffer (100 mM NaCl, 100 mM Tris-HCl, pH 9.5, 50 mM MgCl_2_, 0.1% Tween 20). The alkaline phosphatase chromogen reaction was performed in NTMT buffer containing 100 mg/ml nitroblue tetrazolium (Roche) and 50 mg/ml 5-bromo-4-chloro-3-indolyl phosphate (Roche) at room temperature for 1–7 days and stopped with PBS. Sections were counterstained using nuclear fast red and dehydrated in graded ethanol solutions, and coverslipped with Vectamount (Vector Laboratories). Images were acquired with a Coolsnap CCD camera mounted on a Provis Olympus Microscope.

### Immunohistochemistry and antibodies

For studying cell death, serial sections from 3 WT and 3 KO pups at P2 were immunostained with rabbit anti-activated caspase-3 (1/500 dilution, #559565, BD Pharmingen, France). Sections were permeabilized with PBS-0.25% Triton-X100 (PBS-T) and bathed in blocking solution (PBS-T with 5% normal goat serum). Sections were incubated with the primary antiserum overnight at 4°C, then with Alexa-568 conjugated goat anti-rabbit antibody (1/600, Life Technologies, France) at room temperature for 1 hr. After several rinses, sections were counterstained with DNA dye bisbenzamide (Hoechst 33342, Sigma; 1 µg/ml), and coverslipped under Fluoromount-G (SouthernBiotech, Birmingham, AL, USA).

Caspase-3 positive cells were counted in CA1 and CA3 regions of the dorsal hippocampus across a surface of approximately 5×10^6^ (CA1) and 2×10^6^ (CA3) µm^2^. A 40× objective mounted on a LEICA DM6000 microscope was used for manual counting. Surface area was calculated on corresponding Hoechst images using ImageJ software. 7 sections (each 192 µm apart) per animal were analyzed in a blind manner. The density of caspase-3 positive cells was calculated by dividing their number by the corresponding area. Average densities per animal were estimated and compared between groups with a Student *t*-test using the GraphPrism software.

A panel of different neural precursor marker antibodies were used: rat anti-Pdgfra (1/800, BD biosciences #558774) as an oligodendrocyte precursor marker, rabbit anti-GFAP (1/1000, Dako #Z0334) and rabbit anti-Ndrg2 (1/500; kind gift from T. Miyata, ref [43]) as astrocyte markers, rabbit anti-Pax6 (1/500, Abcam, #ab5790), rabbit anti-Sox2 (1/500, Millipore AB5603) and mouse anti-Nestin (1/500, Millipore MAB353) as more immature precursor markers. Corresponding secondary antibodies coupled to Alexa-488, Alexa-568 and Alexa-647 (1/1000, Life Technologies, France) were used for different antibody combinations. Immunofluorescence was visualized with a Zeiss AxioImager Z1-Apotome system microscope. Pictures were taken with a 20X objective as stacks of 3 to 5 µm with 0.5 µm between sections. Z-projections were done in ImageJ.

### Laser capture microdissection (LCM) and quantitative PCR

To prepare RNA from the CA3 pyramidal cell region, laser microdissection was performed. Coronal brain sections (12 µm) containing the rostro-caudal hippocampus from *Dcx* KO and WT mice were prepared using a cryostat at −20°C and mounted on PENmembrane slides (1440–1000, PALM, Bernried, Germany). Sections were stained with cresyl violet 1% (Sigma Aldrich, France). In WT animals, the single CA3 pyramidal cell layer, incorporating CA3a (closest to CA1) and b (central CA3) regions, was microdissected (Zeiss LCM system), excising at the border with the *strata oriens* and *radiatum.* For *Dcx* KO animals the same region was dissected, however the internal pyramidal cell layer (SPI, closer to the *stratum radiatum*) was excised separately from the external layer (SPE, closer to the *stratum oriens*). For each animal, the entire dorsal hippocampal CA3 structure (bilateral) was excised from 60 sections and pooled.

Total RNA was isolated using a picopure RNA isolation kit (Arcturus) according to manufacturer's instructions. RNA quantity and quality were assessed using a Nanodrop ND-1000 spectrophotometer (ThermoScientific) and Agilent 2100 Bioanalyzer (Agilent Technologies, USA) respectively. To generate cDNA for qPCR experiments, RNA reverse transcription and amplification was performed using a Nugen Ovation Pico WTA system following manufacturer's instructions. Real time qPCR assays using the SYBRgreen method followed MIQE guidelines [44]. Gene-specific primers for *Olig*1 (forward CGTCTTGGCTTGTGACTAGCG and reverse GCCAGTTAAATTCGGCTACTGTC), for *Npy* (forward GCTCTGCGACACTACATCAATCTC, reverse AGGGCTGGATCTCTTGCCATA) and for *Sst* (forward GGAAGACATTCACATCCTGTTAGCTT reverse AGAGGTCTGGCTAGGACAACAATATTA) were designed using Primer Express Software (PE Applied Biosystems). Values were normalized to the geometric mean of 3 Normalization Factors found to be stable in all samples using the geNorm approach (http://medgen.ugent.be/~jvdesomp/genorm/). These were ATP synthase, H+ transporting mitochondrial F1 complex, beta subunit (*Atp5b*), eukaryotic translation initiation factor 4A2 (*Eif4a2*) and prosaposin (*Psap*). For test genes, average values ± standard deviations were calculated for at least 4 animals of each genotype. Ratios of *Olig*1 and *Npy* gene expression fold changes for SPI versus WT, and SPE versus WT were calculated, and *P* values obtained using the Student *t*-test.

### Electron microscopy (EM) and morphometry

Semi-thin sections (0.5 µm) were stained with toluidine blue and images were acquired with a Coolsnap CCD camera mounted on a Provis Olympus Microscope. Ultra-thin sections (40 nm) were mounted on either 200 mesh or one slot grids, cut and double stained with uranyl acetate and lead citrate prior to observation with a Philips (CM-100) electron microscope. Digital images from the CA3 region were obtained with a CCD camera (Gatan Orius).

Morphometric analysis was performed with the software Digital Micrograph. For each nucleus, its widest diameter was measured, and the mean calculated from 50 neuronal nuclei from two mice per genotype. Only neurons displaying a clearly visible nucleolus were taken into account. Golgi apparatuses (GA) were analyzed in 50 cells from two WT animals and two KO animals. Altered versus normal mitochondria were counted in a minimum of 20 WT, 20 SPI and 20 SPE cells from 4 KO mice.

## Results

### EM characterization of the two *Dcx* KO layers in the P0 CA3 region

**Figure 1:**
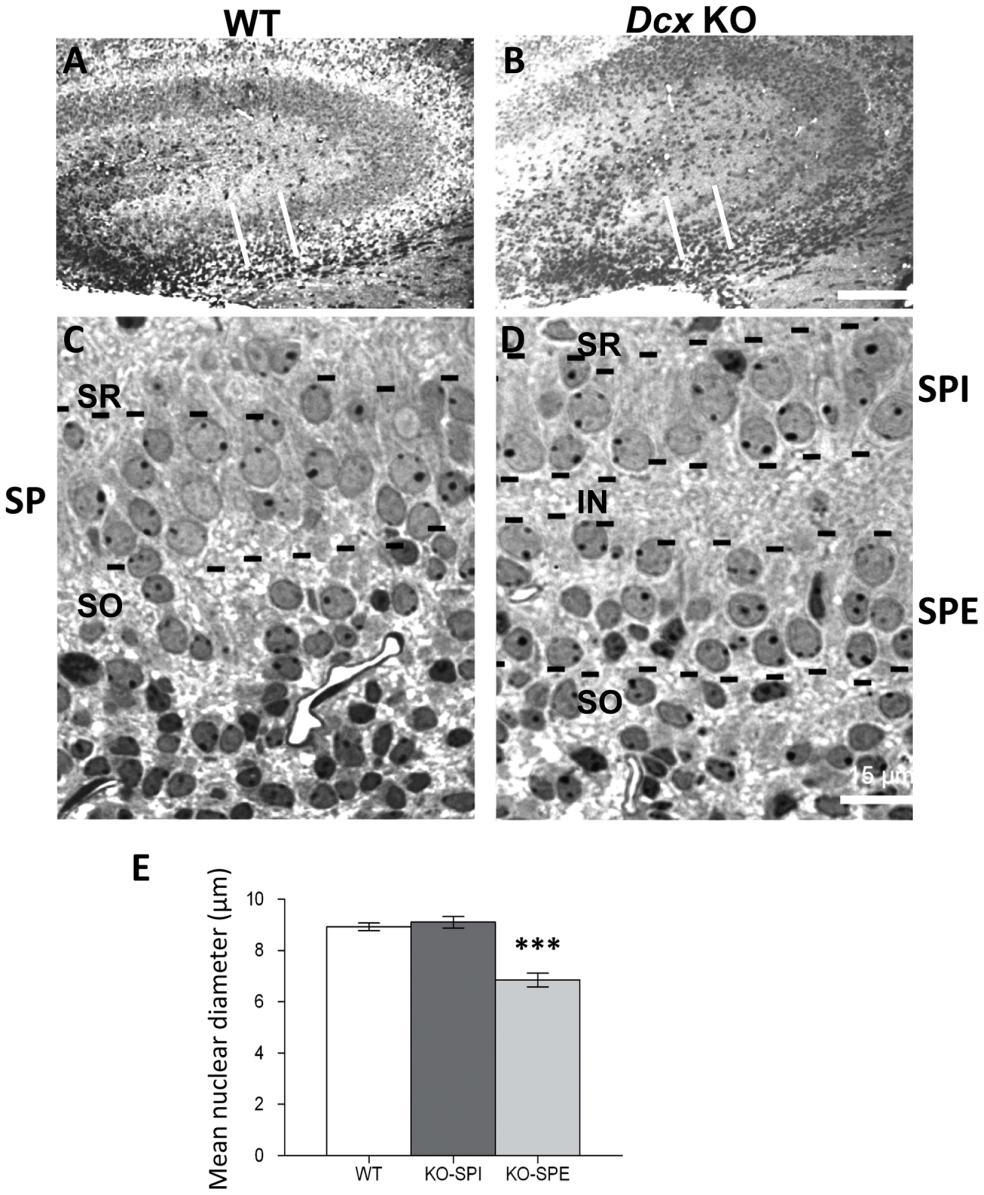
Morphological features of the two *Dcx* KO CA3 neuronal layers at P0. (A–D). Coronal semi-thin sections of the CA3 hippocampal region in wild-type (A, C) and KO (B, D) mice. (A, B) Low magnifications showing the CA3 hippocampal region. The area encompassed by the two vertical white lines indicates the region visualized in C and D. (C, D) Higher magnifications showing nuclei in the CA3 pyramidal layers (delimited by black dotted lines). In WT (C) the pyramidal neurons contain nuclei with a similar aspect. In KO (D), two pyramidal cell layers are visualized, an external layer (SPE) closest to the neuroepithelium bounding the *stratum oriens* (SO), and an internal layer (SPI) in the *stratum pyramidale* region but positioned closer to the *stratum radiatum*. They are separated by an intermediary layer (IN) with a neuropil-like aspect. Each KO pyramidal cell layer contains nuclei with different shapes and sizes. Scale bars A, B: 150 µm; C, D: 15 µm. (E) Measurements of nuclear diameter. Nuclear diameters from the SPI and SPE layers in KO mice are significantly different from each other (Mann-Whitney test, *p*<0.0001). SPE nuclei are significantly smaller than WT (Mann-Whitney test, *p*<0.0001) and SPI nuclei not significantly different. Nuclear diameters from WT and KO (not distinguishing between SPE and SPI), also differ significantly (Mann-Whitney test, *p*<0.0001).

Histological studies to characterize the *Dcx* KO hippocampus were previously performed between E17 and P6 [6]. From postnatal stages two distinct layers of pyramidal cells were clearly visible in the CA3 region, continuing in the adult (Fig. S1 and [6], [27]). Despite their abnormal positioning along the radial axis, pyramidal cells appeared correctly specified, expressing appropriate field markers (Fig. S1). We defined the two KO CA3 layers (Fig. 1) as *stratum pyramidale* internal (SPI, inner layer, closest to the *stratum radiatum*) and *stratum pyramidale* external (SPE, outermost layer, closest to the *stratum oriens*).

Well-organized and aligned CA3 pyramidal cell nuclei were identified in coronal semi-thin WT P0 sections (Fig. 1A, C). In the *Dcx* KO sections, the two cell layers (marked by black dotted lines in Fig 1D) were present over a larger, less cell dense region (Fig. 1B, D), separated by a plexiform-like intermediary layer (IN). Cells in the *Dcx* KO SPE layer were distinguished from other cells in the *stratum oriens* by their neuronal nuclear aspect [45] and their contiguous arrangement, without large free spaces that characterized the neuroepithelium (Fig. 1D). Within the WT layer, cells were closely packed and their nuclei displayed round or oval shapes enclosing one or two nucleoli (Fig. 1C). SPE and SPI KO nuclei were in general more widely spaced than those in the WT layer. Nuclei in SPE possessed less regular shapes and were less well aligned than SPI nuclei. The average nuclear diameter was significantly smaller in SPE than in SPI and WT layers (expressed as mean ± standard error WT, 8.92±0.15 µm; SPI, 9.11±0.23 µm; SPE, 6.85±0.26 µm. SPE versus WT p<0.0001; SPI versus WT p = 0.86; Mann-Whitney test, Fig. 1E). Thus the SPE can be distinguished from the SPI at this stage.

**Figure 2:**
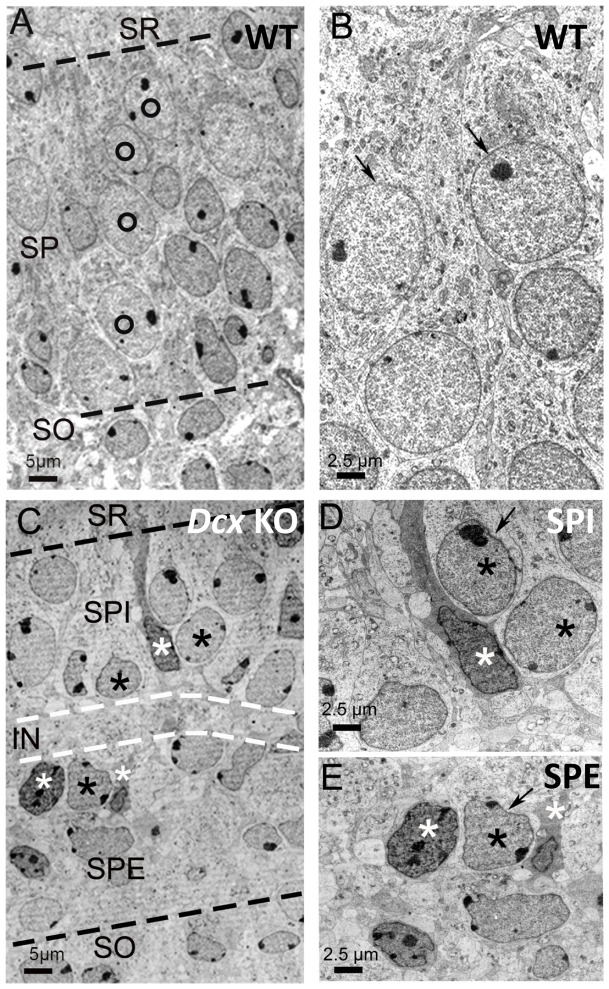
*Dcx* KO CA3 layers show heterogeneous and disorganized cells compared to WT. Electron micrographs of the hippocampus from WT (A–B) and KO mice (C–E). (A) In WT, the broad layer of pyramidal neurons (delimited by dashed black lines) shows a more regular arrangement of adjacent columns of neurons (marked by circles). A layer bordering the *stratum oriens* (SO) displays neurons with occasional oval elongated shaped nuclei. All nuclei are surrounded by a light cytoplasm. (B) High magnification of pyramidal neurons displaying rounded or oval nuclei, with a homogeneous aspect of the nuclear membrane. (C) In the KO, the SPE bounds the SO (lower dashed black line) and the SPI is limited by the *stratum radiatum* (SR) (upper dashed black line). In both the SPE and SPI layers two types of cells are shown. One exhibits a clear cytoplasm and large nuclei (black asterisks) with round, elongated or lobulated shapes, enclosing light chromatin with peripherally located nucleoli, similar in aspect to the neuronal chromatin observed in WT. The second type of cell (white asterisks) has a darker cytoplasm, occasional tapered processes and encloses nuclei with dense chromatin. They are intermingled and often closely juxtaposed with neuronal-like cells. (D) In the SPI, two types of cells are shown, pyramidal neuronal-like cells with a clear cytoplasm and peripheral nucleoli (black asterisk) and a second cell type with a darker cytoplasm whose nucleoli are rarely peripheral (white asterisk). (E) In the SPE similar cell types with light (black asterisk) and dark cytoplasm (white asterisks) are also identified. Arrows in D and E indicate the irregular shape of the nuclear membrane. Scale bars: A, C: 5 µm; B, D, E: 2.5 µm.

At higher magnification (Fig. 2A), the broad single WT pyramidal cell layer showed superposed layers of neuronal-like cells (Fig. 2B, black circles), characterized by a clear abundant cytoplasm (Fig. 2B). *Dcx* KO CA3 layers contained some morphologically different cell types from those observed in WT. A first cell type showed clear cytoplasm and round or elongated nuclei (Fig. 2C, black asterisks) enclosing light chromatin and peripherally located nucleoli, similar in aspect to neuronal-like cells in WT. The *Dcx* KO neuronal-like nuclei sometimes showed an irregular or lobulated form compared to WT (Fig. 2 compare D, E to B), and clumped chromatin (data not shown). A second cell type with darker cytoplasm (white asterisks in Fig. 2C) was observed interspersed between neuronal-like cells in both SPE and SPI. Such cells in some cases displayed processes, occasionally surrounding neuronal-like cells (Fig. S2). Their nuclei were often smaller than those of neurons, and showed irregular contours and dark chromatin, with one or several nucleoli. Thus, more heterogeneous cellular forms were observed in *Dcx* KO CA3 layers compared to WT.

### Organelle abnormalities identified in *Dcx* KO neuronal cells

**Figure 3:**
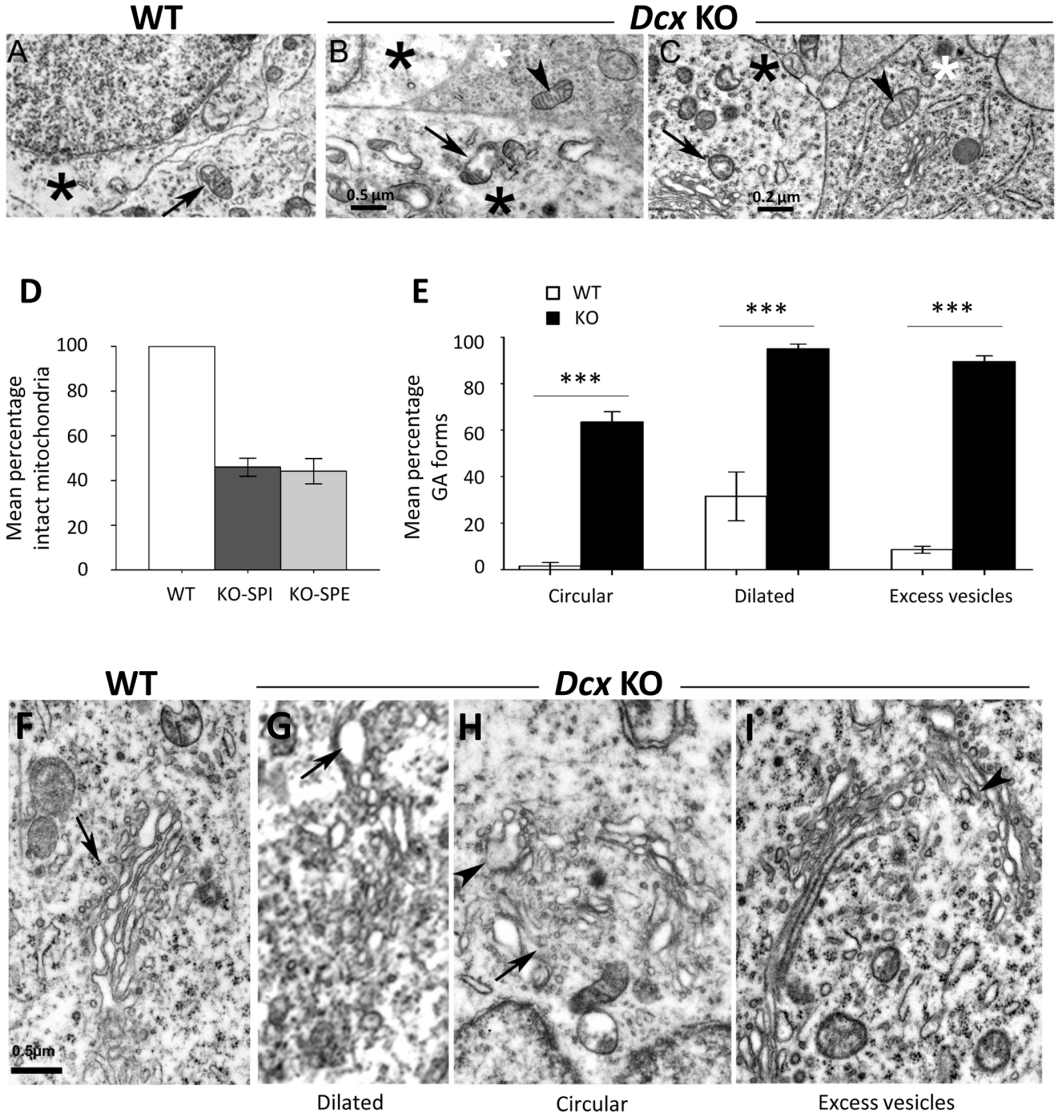
Ultrastructural organization of *Dcx* KO SPI and SPE cells, showing mitochondria and Golgi apparatuses. Electron micrographs of the hippocampus from WT (A, F) and KO (B, C, G–I). (A) High magnification of a WT pyramidal neuron showing its cytoplasm (black asterisk) and the cytoplasm of a neurite enclosing a normal mitochondrion (arrow). (B, C) Details of two juxtaposed KO neuronal-like cells (recognized by their lighter cytoplasm, black asterisks) with altered mitochondria (arrows), compared to a cell with darker cytoplasm (white asterisks), exhibiting a normal mitochondria (arrowheads) and Golgi apparatus. (D) Quantitative analyses of the intact mitochondria in WT and *Dcx* KO hippocampal CA3 cells. (E) Quantitative analyses of the modified Golgi apparatus forms between WT and *Dcx* KO hippocampal CA3 cells. Mean percentage of the detected abnormality for each genotype. Note significant differences between the genotypes for each form of Golgi (Fisher exact test, *p*<0.0001). (F) A typical Golgi apparatus (arrow) is observed in a WT pyramidal neuron. This consists of a parallel array of tubules and cisternae with few vesicles. (G–I) Golgi apparatuses in KO cells showing the heterogeneous aspect of the cisternae. Scale bars: A, B, 0.5 µm; C, 0.2 µm; F–I: 0.5 µm.

In WT cytoplasm, mitochondria were found to have a normal aspect, with clearly defined cristae (arrow in Fig. 3A), whereas *Dcx* KO neuronal-like mitochondria were often altered and swollen (arrow in Fig. 3B). Both normal and altered mitochondria were found in the same cell, including in neuritic processes. On the other hand, in darker cells closely juxtaposed to *Dcx* KO neuronal-like cells, mitochondria possessed well defined cristae (arrowheads, Fig. 3B, C). Numbers of damaged mitochondria were further assessed in a number of randomly selected neuronal somata. No abnormal mitochondria were observed in WT cells (n = 20 cells). However, neuronal-like KO cells (n = 21 SPE, n = 28 SPI) showed only 45% normal mitochondria, similar in SPI and SPE (Fig. 3D). Thus, mitochondrial abnormalities are present in the *Dcx* KO CA3 region and they appear to be cell-type specific, restricted to neuronal-like cells.

*Dcx* KO Golgi apparatuses also appeared modified compared to WT (Fig. 3E–I), exhibiting increases of cisternae with circular disposition (found in 62% of cells, only 2.2% for WT, Fisher's exact test *p*<0.0001), of terminal dilated cisternae (96.5% of cells, 33.3% for WT, Fisher's exact test *p*<0.0001), and of the presence of more than 10 Golgi vesicles (91,4% of cells, 8.9% in WT, Fisher's exact test *p*<0.0001). Thus, as well as mitochondrial abnormalities, *Dcx* KO neuronal-like cells exhibit Golgi apparatus differences, with juxtaposed, darker cells apparently unaffected (Fig. 3C).

### Characterization of the intermediary (IN) layer separating the SPI from the SPE

**Figure 4:**
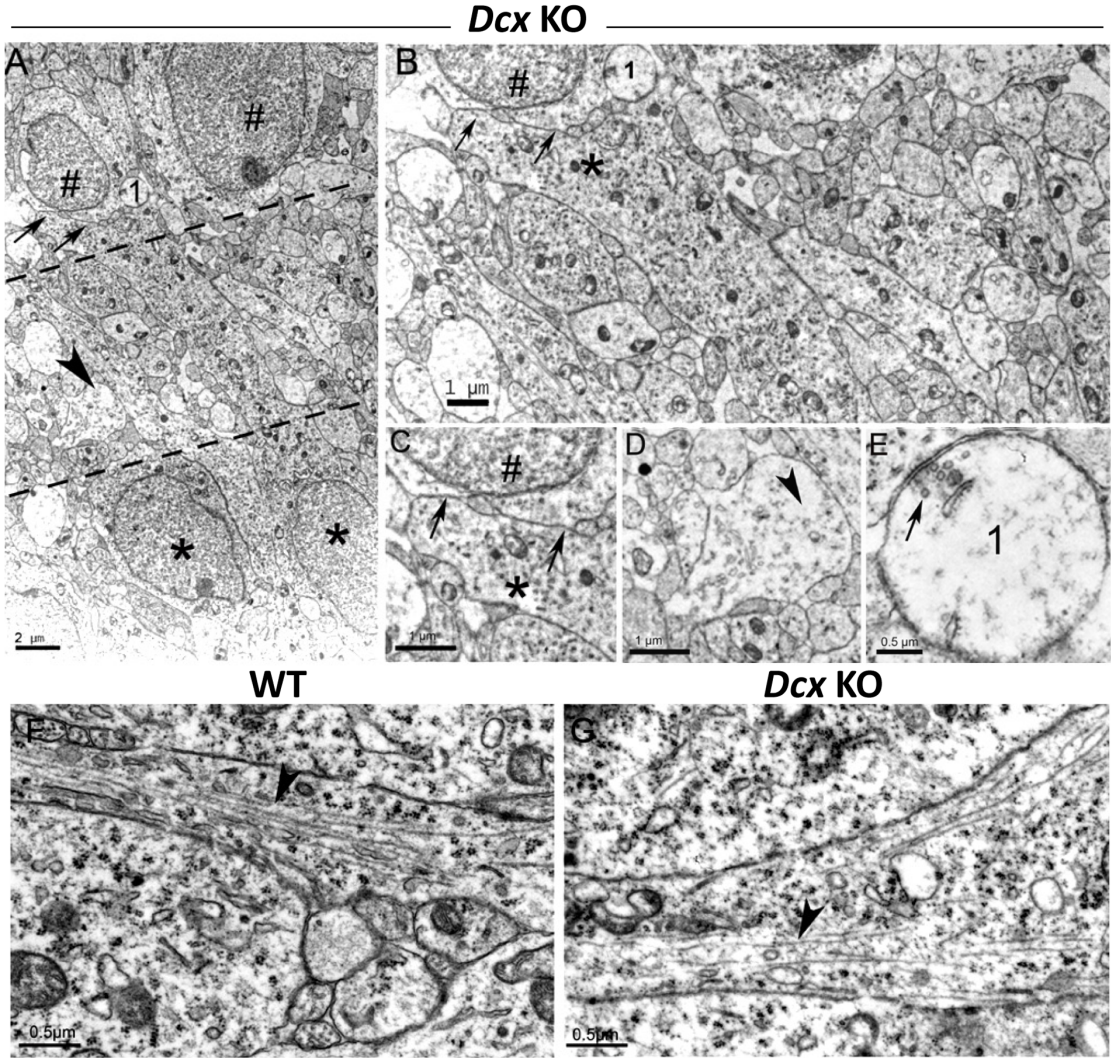
Ultrastructural organization of the intermediary layer (IN). (A) Low magnification showing the IN delimited by black dashed lines. Cells from the SPI (black symbols #) and SPE (black asterisks) flank numerous neuritic profiles enclosed in the IN layer. The arrows, arrowhead and ‘1’ correspond to zones enlarged in C–E respectively. (B) Higher magnification of the IN. Numerous dense cellular profiles are visualized, corresponding to neuronal processes (likely to be immature dendrites and axons) and synaptic contacts (see ‘E’). A neurite from a cell located in the SPE (black asterisk) extending to and juxtaposing (arrows) the soma from a cell in the SPI (black symbol #). (C) High magnification of the neuropil-like region indicated by two arrows in A and B. Note the close apposition between a probable dendritic profile from an SPE cell and an SPI soma (black symbol #). (D) Higher magnification of arrowhead in A. The cellular profiles of the IN display heterogeneous aspects: some of them with a light cytoplasm correspond to transversal sections of neurites, and contain tubulo-vesicular structures and regions without cytoskeletal elements (arrowhead). (E) is a detail of an axo-somatic synaptic contact identified in A and B as ‘1’. Some synaptic vesicles are observed (arrow). (F, G) Neurites of hippocampal pyramidal neurons. (F) A neurite from a WT pyramidal cell, longitudinally sectioned, contains numerous adjacent microtubules closely arranged in parallel fascicles (arrowhead). (G) In KO, a neurite from an SPI pyramidal neuron shows sparser, more widely-spaced microtubules (arrowhead). Scale bars: A, 2 µm; B, C, D: 1 µm; E–G: 0.5 µm.

The *Dcx* KO IN layer was found to contain numerous heterogeneous, neuritic profiles (Fig. 4A–E). Nuclei were largely absent from this region. Neuritic profiles were not oriented uniformly and displayed heterogeneous forms, ranging from round to elongated (Fig. 4B). Some displayed a light cytoplasm with damaged mitochondria (Fig. 4C), and contained tubulo-vesicular structures (Fig. 4D) and regions devoid of cytoskeletal elements (arrowhead, Fig. 4D). Microtubules in longitudinally sectioned profiles, when obvious in *Dcx* KO neurites, appeared less numerous than those in WT cells (compare Fig. 4F, G).

Some neuritic contacts were observed, for example, between *Dcx* KO apical neurites (black asterisk, Fig. 4C) emerging from a neuronal cell located in the SPE and extending to a neuronal soma in SPI (as shown in Fig. 4A, B). This profile could represent a growing apical dendrite. Synaptic contacts were also occasionally observed at the border of the IN, in some cases likely to be immature symmetric synapses of GABAergic cells, contacting perisomatic regions of pyramidal cells (contact with an SPI soma shown in Fig. 4B, E). They contained a few synaptic vesicles (arrow) opposite the postsynaptic zone. These data indicate that, despite abnormal cellular profiles, synaptogenesis can take place at P0 in *Dcx* KO neuronal-like cells.

### Heterogeneous cell types in close proximity to *Dcx* KO pyramidal cells

We set out to identify the heterogeneous cell types revealed by EM, interspersed within, or in close proximity to, the *Dcx* KO pyramidal cell layers. Anti-Pax6 and anti-Sox2, two markers of apical radial glial cell progenitors [46], normally present in the VZ during hippocampal development, were first used. Pax6- and Sox2-positive nuclei were only seen in their correct position in the VZ of both WT and KO brains and were not present in the pyramidal layers (Fig. S3A–F). Similarly, we used anti-nestin, which labels intermediate filaments in radial glial cell progenitors, including their processes. There was no evidence of nestin-positive somata within the pyramidal cell layers in either WT or KO (Fig. S3C–F). GFAP, a marker of radial glial cells and mature astrocytes, also showed no labeling at this stage in the pyramidal cell layer in either WT or KO hippocampi (Fig. S3G, H). Therefore the cellular heterogeneity revealed by EM is not due to abnormal positioning of radial glial progenitors or astrocytes.

**Figure 5:**
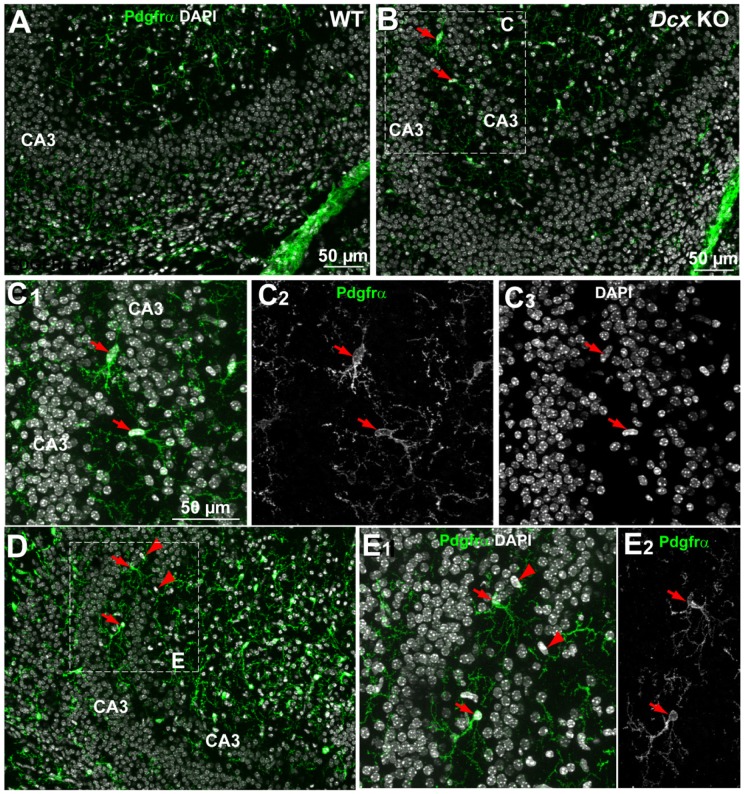
Oligodendrocyte precursor cells (OPCs) in WT and *Dcx* KO CA3 regions. Immunoreactivity of Pdgfrα (to detect OPCs) observed in the CA3 region in P0 WT (A) and *Dcx* KO (B–E). The green fluorescent immunolabelling corresponds to OPCs whereas DAPI staining reveals cell nuclei (white). No Pdgfrα^+^ cells are revealed in the pyramidal cell layer in WT (A). In the KO, the red arrows (B–E) indicate the presence of Pdgfrα^+^ cells, characterized by ovoid nuclei intermingled among CA3 neurons. Arrowheads in D, E show other ovoid nuclei in the CA3 region of the *Dcx* KO that are Pdgfrα-negative. C and E are higher magnifications of the areas in the insets of B and D, respectively. Scale bars: 50 µm.

Platelet-derived growth factor receptor α (Pdgfrα), a marker of OPCs, was next tested. In the WT CA3 region, cell somata were present in the *strata oriens* and *radiatum* but rarely in the pyramidal cell layer (Fig. 5A). However, a number of OPCs were identified within and adjacent to the *Dcx* KO pyramidal cell layers (Fig. 5B–E and Fig. S3). OPC nuclei were either horizontally, or vertically oriented (Fig. 5C1-3, E1-2 arrows) and were well arborized. In order to confirm the higher abundance of OPCs in the *Dcx* KO *stratum pyramidale* compared to WT, quantitative PCR (qPCR) was performed for the OPC marker, *Olig*1 (Table 1 and Table S1). We found a two-fold up-regulation of this transcript in *Dcx* KO SPE compared to WT (Student *t*-test, *p* ≤0.001) and a five-fold up-regulation in *Dcx* KO SPI compared to WT (Student *t*-test, *p*<0.01). These combined results suggest a significant excess of OPCs interspersed within the P0 *Dcx* KO pyramidal cell layers.

**Table 1:** qPCR results (mean expression values, expressed in relative units) for *Olig*1 and *Npy*.

Gene	Expression WT	Expression SPI	Expression SPE	*p* WT versus SPI	*p* WT versus SPE
*Olig*1	0.031±0.01	0.16±0.01	0.060±0.01	0.008	0.001
*Npy*	0.014±0.005	0.05±0.03	0.027±0.02	0.008	0.118

**Figure 6:**
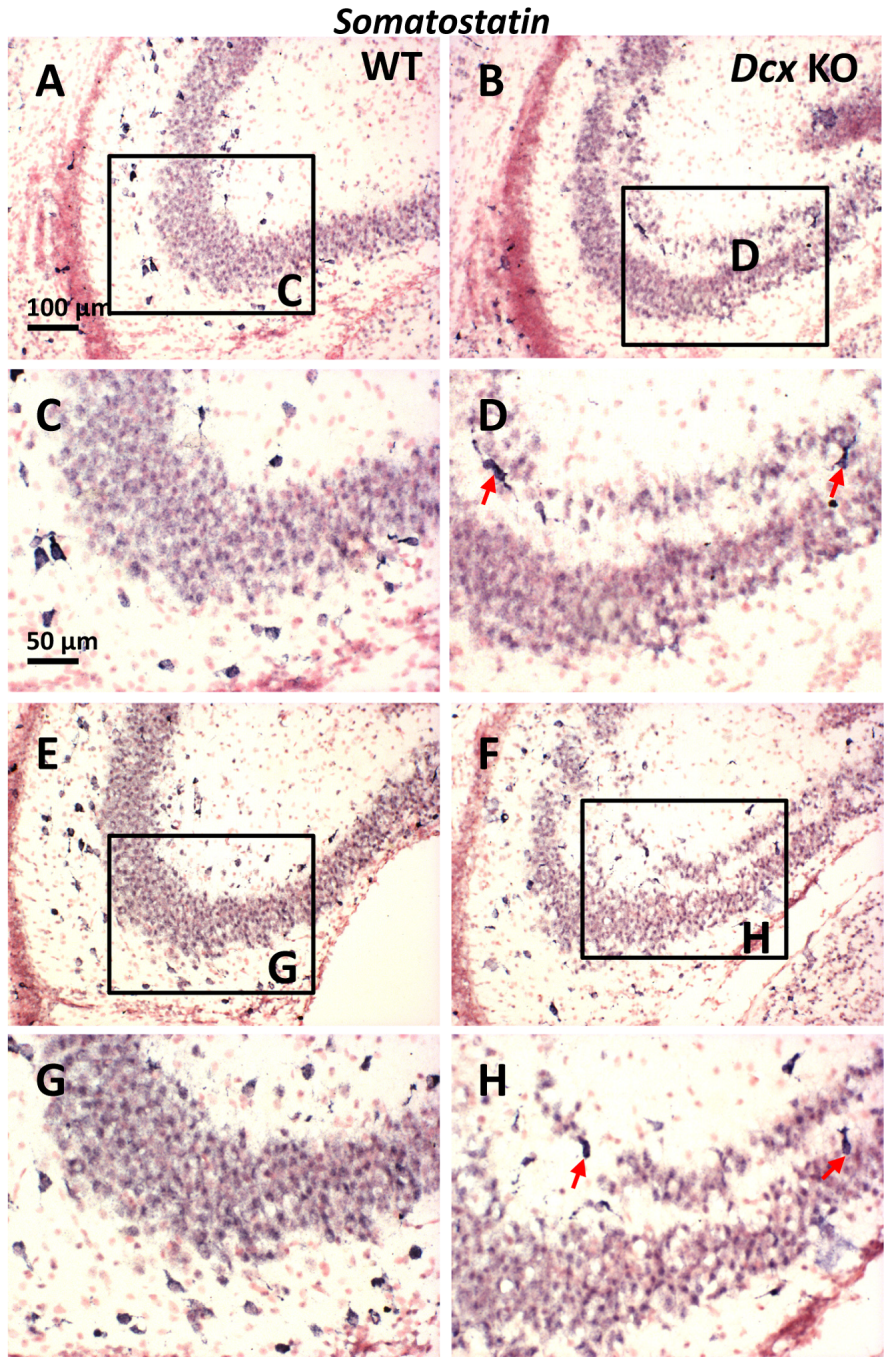
*Sst* interneurons in the region of the *stratum pyramidale* of WT and *Dcx* KO hippocampi. (A–H) Immunoreactivity to *Sst* transcripts in WT (A, C, E, G) and KO (B, D, F, H) mouse brain coronal sections. Representative images showing *in situ* hybridization results at two levels of the dorsal hippocampus (A–D; E–H). C, D and G, H are higher magnifications of A, B and E, F respectively. In WT, *Sst*-positive interneurons are mainly found outside the *stratum pyramidale* in the *stratum oriens* and *radiatum* at this age. In the *Dcx* KO, some *Sst*-positive cells are observed associated with the SPI, IN and occasionally the SPE. Scale bars, A (for A, B, E, F) 100 µm; C (for C, D, G, H) 50 µm.

Further unlabeled nuclei were also observed in immunohistochemistry experiments, some potentially with an aspect of migrating cells based on their vertically oriented, oval shaped nuclei (Fig. 5D, E1, red arrowheads). These cells could correspond to migrating pyramidal cells or interneurons, or other cell types. Concerning interneurons, we have previously assessed the number of parvalbumin positive cells in the adult *Dcx* KO hippocampus, showing no overall differences in number compared to WT [27]. Somatostatin (Sst)-positive cells represent a second major subpopulation [47], which can co-express either reelin, neuropeptide Y (Npy) or calretinin markers [48]. This subpopulation is present in the CA3 region perinatally, but not normally associated with the *stratum pyramidale* and we hence decided to test for these cells in *Dcx* KO pyramidal layers. At early postnatal stages in WT, *Sst*-interneurons were found mainly in the *stratum oriens,* with some somata also found in the *stratum radiatum* (Fig. 6A, C, E, G) and in the hilus (data not shown). In the *Dcx* KO, as well as being observed in these regions, some *Sst*-positive cells were also found associated with the SPI, SPE or the IN region (Fig. 6B, D, F, H). Such cells were vertically, obliquely or horizontally oriented and some showed a potentially migratory morphology (Fig. 6H, arrows). Similarly, qPCRs for *Npy* showed an upregulation especially in the SPI layer compared to WT (3.6 fold up-regulation, Student *t*-test, *p*≤0.01, Table 1 and Table S1), although the *Sst* transcript tested was not found significantly different in this assay. These data together suggest that, as well as OPCs, certain interneuron populations or their migrating precursors may also contribute to cell heterogeneity observed at P0 in the *Dcx* KO pyramidal layers.

### Cell death is increased in the *Dcx* KO hippocampus

**Figure 7:**
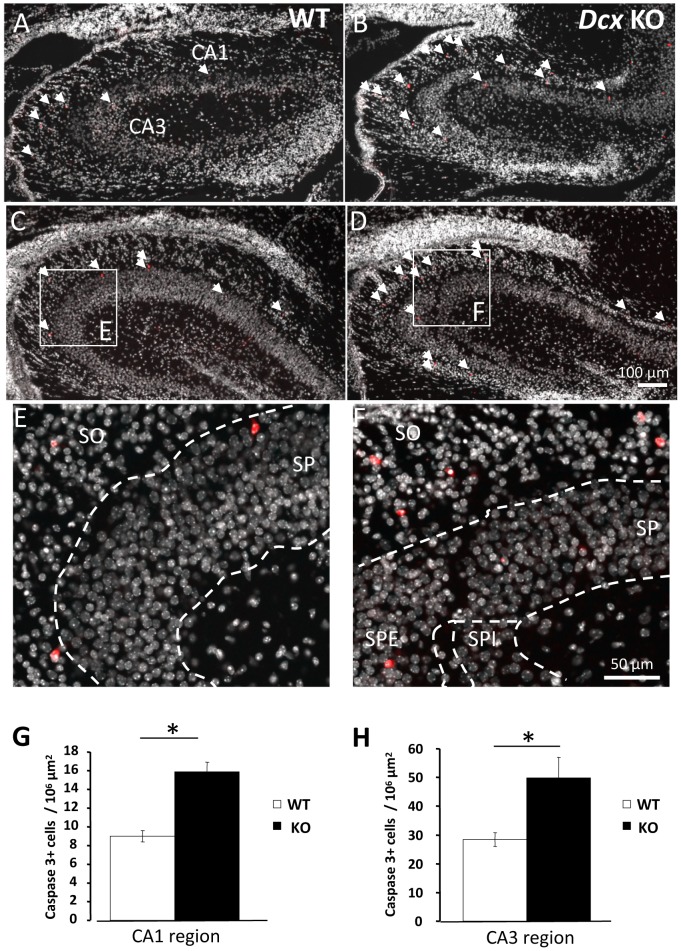
Apoptosis is increased in the postnatal *Dcx* KO hippocampus. Immunoreactivity to activated caspase-3, and Hoechst staining observed in P2 WT (A, C, E) and *Dcx* KO (B, D, F) dorsal hippocampi at rostral (A, B) and more caudal (C, D) hippocampal levels. Arrowheads in A–D indicate the positions of labelled cells, which had typical features for apoptotic cells. E and F are higher magnifications corresponding to the inset regions in C and D. Caspase-3 positive cells are present in both the *stratum oriens* (SO) and some in the *stratum pyramidale* (SP) of the CA3 region of both WT (E) and KO (F) hippocampi. (G, H) Histograms represent the cellular density of activated caspase-3 expressing cells in CA1 and CA3 regions respectively of WT versus KO hippocampi. Note the increase in activated caspase 3-positive cells in the *Dcx* KO, *p*<0.05. Scale bars: A–D: 100 µm; E–F: 50 µm.

The abnormalities identified in the *Dcx* KO hippocampus also prompted us to assess cell death. We examined apoptosis at P2, a peak stage of cell death during rodent postnatal hippocampal development [37], [38]. Cells expressing activated caspase-3 were identified across the rostro-caudal extent of the *Dcx* KO dorsal hippocampus compared to WT littermate hippocampi. At this postnatal stage in both WT and KO, activated caspase-3 labeled cells were present at highest densities in the CA3 region as well as to a lesser extent, in the CA1 region (Fig. 7A–D, white arrowheads). In both CA3 and CA1 regions, caspase-3 positive cells were predominantly present in the *stratum oriens*, although some cells could also be seen in the *stratum pyramidale* (Fig. 7E–F). Analyzing CA1 and CA3 regions separately, an approximately two-fold increase of caspase 3-positive cells was present in each hippocampal region in the KO compared to WT (Fig. 7G–H, [in CA1 9 cells/10^6^ µm^2^ in WT versus 15.8 cells/10^6^ µm^2^ in *Dcx* KO, *p*<0.05; in CA3 28.3 cells/10^6^ µm^2^ in WT versus 49.8 cells/10^6^ µm^2^ in *Dcx* KO, *p*<0.05]). Thus overall, higher numbers of caspase-3 positive cells were found across the rostro-caudal extent of postnatal hippocampi from *Dcx* KO animals, a result which also highlights the abnormal development of this region.

## Discussion

We characterized here the state of the *Dcx* KO hippocampus using EM, immunohistochemistry and qPCRs. We found the developing *Dcx* KO pyramidal layers to be quite different from WT, being less organized and more heterogeneous. Organelle abnormalities were identified in *Dcx* KO neuronal-like cells, which has not previously been shown to our knowledge in other heterotopia mouse models. Indeed, few previous studies have addressed ultrastructural aspects of abnormally positioned neurons by EM, although analyzes of the highly disorganized embryonic *reeler* cortex revealed cell mis-orientation with grossly normal neuronal differentiation [49]. Our postnatal data, potentially illustrating early consequences of abnormal neuronal position, provide a framework on which to study how functional abnormalities arise in the *Dcx* KO hippocampus, especially the development of cell hyperexcitability [26], [27].

In WT embryos, the peak of neurogenesis for the CA3 region occurs at E14 [50], although some CA3 pyramidal cells are still produced at E16 [35]. Migration occurs across a 4 to 5 day period before cells reach the hippocampal plate, after pausing extensively in the IZ [25], perhaps waiting for coordinating signals from interneurons and dentate gyrus granule cells [31], [33]. We looked at the state of cells in early postnatal development, thus at a time when migration is expected to be largely completed [25], [28]. The presence and position of two *Dcx* KO layers, even from E17.5 [6], suggests slowed or arrested migration, with SPE cells potentially remaining displaced even in the adult [6], [22]. We hence searched for differences between the SPE and SPI layers. Indeed, we found that SPE neurons showed a relatively smaller nuclear diameter than SPI neurons at P0. This may be related to the fact that they are less mature [51], [52], since a previous study of immature hippocampal neurons in foetal monkey brain showed that as migrating pyramidal cells reach their final position they become progressively more complex, with larger somata and less electron-dense nuclei [51]. SPE cells at P0 may hence be less mature than SPI and WT neurons, and perhaps retarded or arrested in their migration. The additional observation of increased cell death at P2 in the *Dcx* KO CA3 region, in particular in the *stratum oriens*, may also be linked to the elimination of some aberrant cells arrested in their migration.

We identified some oval-shaped nuclei within both *Dcx* KO layers, which may correspond to some still-migrating pyramidal cells, interneurons, OPCs, or other cell types. Indeed, probable migratory elongated somata, associated with lobulated nuclei containing relatively dense chromatin, and tapered leading processes, were previously characterized by EM in the developing mouse brain by Pinto-Lord and colleagues [53]. Based on the identification of similar cells in our EM study, this reinforces the idea that a certain number of cells continue to migrate in the postnatal *Dcx* KO hippocampus.

Despite the possible immaturity of the SPE layer, some synaptic contacts were clearly being formed. Indeed, the IN layer enclosing different types of cellular profiles resembles a plexiform layer, likely to contain growing axons, and apical and basal dendrites of SPE and SPI pyramidal cell neurons respectively, as well as incoming mossy fibers. We previously showed by biocytin labeling that adult SPE apical dendrites can cross the SPI [27]. Neurites from the SPE, observed by EM in this study to extend up to and to contact SPI cells, may be, at least in part, such dendrites. Interestingly, mature apical dendrites of SPE cells and basal dendrites of SPI cells in the adult KO hippocampus showed reduced length compared to WT [27]. Stunted dendrite growth in the IN might hence be a consequence of the bi-layered organization. On the other hand, a bi-laminar organization of CA1 apparently naturally exists in the wallaby, possum and mole rat, although in this case, only the inner, superficial layer is compact, whereas the ‘outer layer’ represents a continuum of sparser cells [54]. Differing from this situation, already at P0, *Dcx* KO SPI and SPE cell layers seem relatively compact and can be distinguished from other cells in the *stratum oriens,* likely to be migrating granule cell precursors [28]. Thus the divided pyramidal cell layer in the *Dcx* KO does not appear to resemble other naturally existing and well-functioning hippocampal ‘bi-layers’.

Although *Dcx* KO cells are relatively condensed in layers, their extracellular environment may still be permissive or attractive for colonization by OPCs and other cell types, which insert themselves into the intercellular spaces. Darker cells identified by EM may thus in some cases represent OPCs. These cells, which displayed normal organelles, may not normally express Dcx, which is largely restricted to the neuronal lineage [55], [56]. Developing OPCs are proliferative cells that originate in the ventral telencephalon and then migrate throughout the CNS [57]–[59]. They rapidly increase in number in the developing central nervous system between P0 and P7 and migrate extensively to populate the developing hippocampus before converting into differentiated oligodendrocytes [32]. The final fate of the *Dcx* KO OPCs associated with the pyramidal cell layers at P0, is not yet known. Signaling between *Dcx* KO CA3 pyramidal neurons and OPCs [60], or aberrantly positioned interneurons, could perturb neurotransmission and the development of hippocampal circuits.

Organelle abnormalities were identified in immature *Dcx* KO neuronal-like cells, and to our knowledge, abnormal mitochondria have not previously been reported in classical cortical malformation models, although they have been associated with Zellweger syndrome [61], and more commonly in models of neurodegenerative disorders [62]. Mitochondrial and Golgi abnormalities may stem from aberrant functioning of the microtubule network leading to organelle transport problems [63]. Dcx is a microtubule-associated protein [55], [64], and microtubule abnormalities were previously characterized in *Dcx* KO neurons [65]. As well as direct microtubule functions [66], [67], Dcx may also link organelles to microtubules, indeed it has been previously shown to interact with adaptor complexes, involved in vesicle and cargo trafficking [68], to affect the parameters of molecular motor (kinesin)-directed movement [69], [70], and neurons mutant for two Dcx family members also showed abnormalities in axonal transport and synaptic vesicle deficits [71]. Thus, mitochondrial and Golgi apparatus abnormalities identified here could be a reflection of defects in various microtubule-related functions of Dcx.

Alternatively, organelle differences may arise for other reasons, perhaps related to more generalized cell stress, calcium signaling abnormalities [72], perturbed neurotrophic factors [73], or due to increased oxidative stress [61]. It will be important in the future to identify the causes of these defects and to assess the situation in adult *Dcx* KO pyramidal cells. Apoptosis may eliminate severely deficient cells during development, and autophagy may also play a role in their survival [62], allowing them to function in the adult. Further interrogation of these defects may lead to the identification of novel neuroprotective treatment strategies for this type of lamination disorder.

## Supporting Information

Figure S1 **Correctly specified CA fields in the adult** ***Dcx*** **KO hippocampus compared to WT.** In order to first verify that field identity of CA KO cells was appropriately specified, we used adult hippocampal field specific markers in *in situ* hybridization experiments. *In situ* hybridization results show *Wfs*1 (A, B), *Necab*2 (C, D) and *KA*1 (E, F) markers comparing adult WT (A, C, E) and *Dcx* KO sections (B, D, F). *Wolfram syndrome gene* 1 (*Wfs1*) is a CA1 field specific marker [74]–[76]. In the *Dcx* KO hippocampus, we identified mainly normally positioned CA1 pyramidal cells, although some heterotopic cells were identified close to the subiculum, expressing this marker (Fig. S1B, arrows). Disorganized cells in the KO CA3 region did not express *Wfs*1. *N terminal EF calcium binding protein* 2 (*Necab*2), labels the CA2/CA3a region (the part of CA3 closest to CA2) and cells in the outer border of CA3b (the middle portion of CA3, Fig. S1C). This marker also showed a grossly normal pattern in the *Dcx* KO and revealed the region of splitting of the pyramidal cell layer (Fig. S1D, large arrow). Some *Necab*2-labelled cells were also found in the *Dcx* KO SPI (Fig. S1D, short arrow), with fewer present in the outer border of SPE CA3b. *KA*1 (glutamate receptor kainate type 1) is a well-known CA3 cell marker, whose expression is specified early on during hippocampal development [77]. The *KA*1 marker most intensely labels the CA3 region, and the *Dcx* KO double cell layer is labeled with this marker (Fig. S1E, Fs). Scale bar, 60 µm.
(TIF)

Figure S2 **Arrangement of different cell types in the SPE pyramidal layer.** (A) A region of the SPE layer of the *Dcx* KO hippocampal CA3 region showing neuronal-like cells (black asterisks) surrounded by other cell types with darker nuclei (white asterisks) and thin, dark cytoplasm. These latter cells appear to partially surround the neuronal-like cells. Scale bar: 2 µm.
(TIF)

Figure S3 **OPCs but not astrocytes intercalate between CA3 neurons.** (A–H) Coronal sections of the CA3 region of the hippocampus of P0 WT (A) and *Dcx* KO mice (B–H). (A–B) Sections immunostained for Pdgfrα (red), Pax6 (green) showing that Pax6^+^ cells are only present in the ventricular region, but no ectopic Pax6 cells are found in the mutant CA3 SPE and SPI layers. (C–F) Sections immunostained with Sox2 (red), Pdgfrα (green) and Nestin (light blue) showing that no immature precursors are intercalated between CA3 neurons in *Dcx* mutant hippocampus. Note that some Sox2^+^ cells (arrowheads) can be found below the CA3 region as a stream of cells migrating towards the dentate gyrus (C) or in the *stratum radiatum* (E, F arrowheads). E and F are higher magnifications of boxed areas in C and D. (G, H) Sections immunostained with antibodies for astroglial markers NRDG2 (red), GFAP (pink), and OPC marker Pdgfrα (green) showing that only OPCs, but not astrocytes intercalate between the CA3 neurons in *Dcx* mutant hippocampus. Note astrocytes close to the ventricular surface (G, arrowheads). H is a higher magnification of the area in the inset of G. Scale bars: 50 µm.
(TIF)

Table S1 **qPCR results (** ***Olig*** **1,** ***Sst*** **, and** ***Npy*** **) for individual hippocampal samples (WT and KO).**
(XLSX)
