# Supplementary figures and images for: Organelle and Cellular Abnormalities Associated with Hippocampal Heterotopia in Neonatal Doublecortin Knockout Mice
Source: PLoS One. 2013 Sep 2;8(9):e72622. doi: 10.1371/journal.pone.0072622 (PMC3759370; doi:10.1371/journal.pone.0072622)

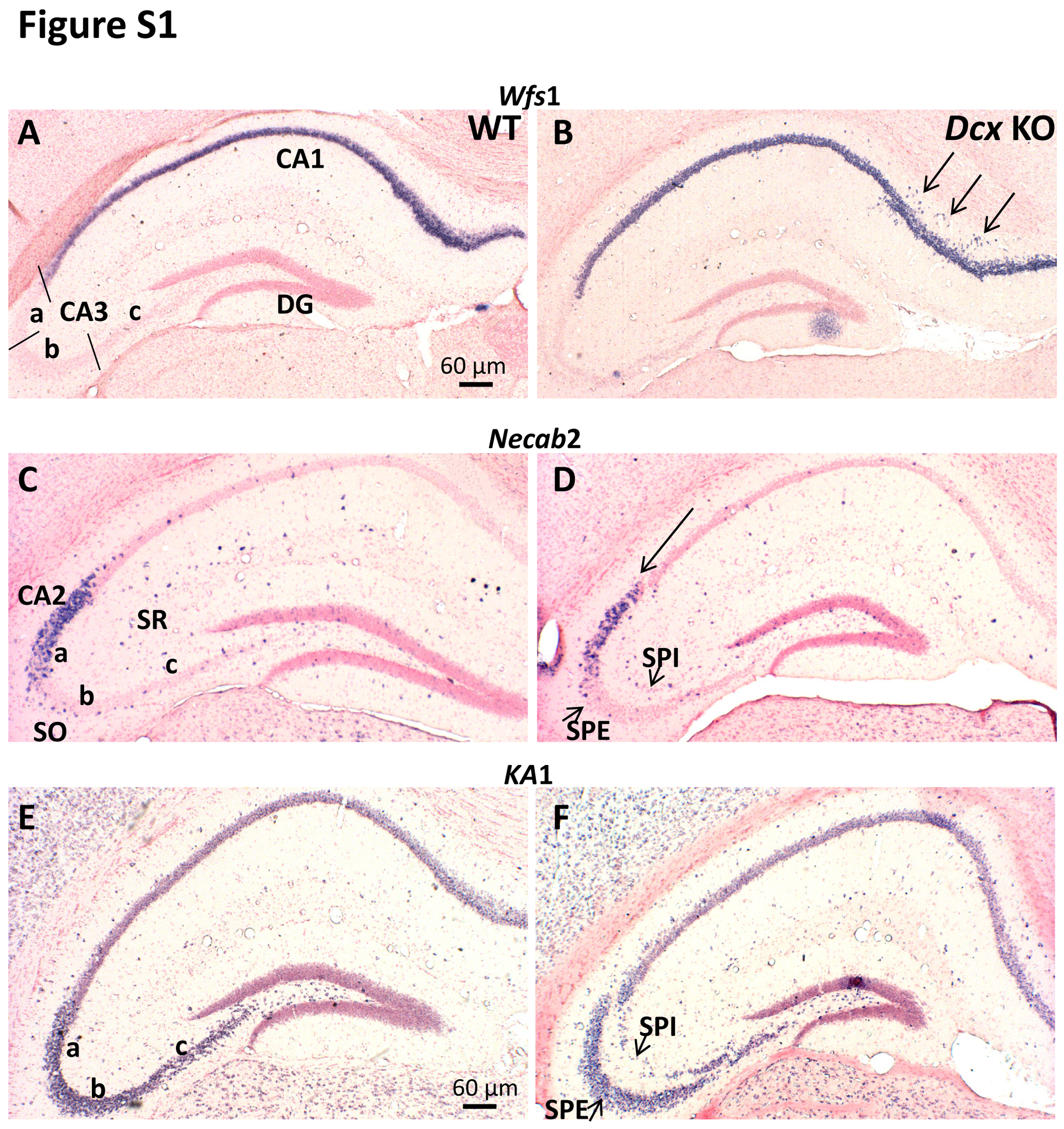

Supplement: Figure S1 — Correctly specified CA fields in the adult Dcx KO hippocampus compared to WT. In order to first verify that field identity of CA KO cells was appropriately specified, we used adult hippocampal field specific markers in in situ hybridization experiments. In situ hybridization results show Wfs1 (A, B), Necab2 (C, D) and KA1 (E, F) markers comparing adult WT (A, C, E) and Dcx KO sections (B, D, F). Wolfram syndrome gene 1 (Wfs1) is a CA1 field specific marker [74]–[76]. In the Dcx KO hippocampus, we identified mainly normally positioned CA1 pyramidal cells, although some heterotopic cells were identified close to the subiculum, expressing this marker (Fig. S1B, arrows). Disorganized cells in the KO CA3 region did not express Wfs1. N terminal EF calcium binding protein 2 (Necab2), labels the CA2/CA3a region (the part of CA3 closest to CA2) and cells in the outer border of CA3b (the middle portion of CA3, Fig. S1C). This marker also showed a grossly normal pattern in the Dcx KO and revealed the region of splitting of the pyramidal cell layer (Fig. S1D, large arrow). Some Necab2-labelled cells were also found in the Dcx KO SPI (Fig. S1D, short arrow), with fewer present in the outer border of SPE CA3b. KA1 (glutamate receptor kainate type 1) is a well-known CA3 cell marker, whose expression is specified early on during hippocampal development [77]. The KA1 marker most intensely labels the CA3 region, and the Dcx KO double cell layer is labeled with this marker (Fig. S1E, Fs). Scale bar, 60 µm. (TIF) [file pone.0072622.s001.tif]

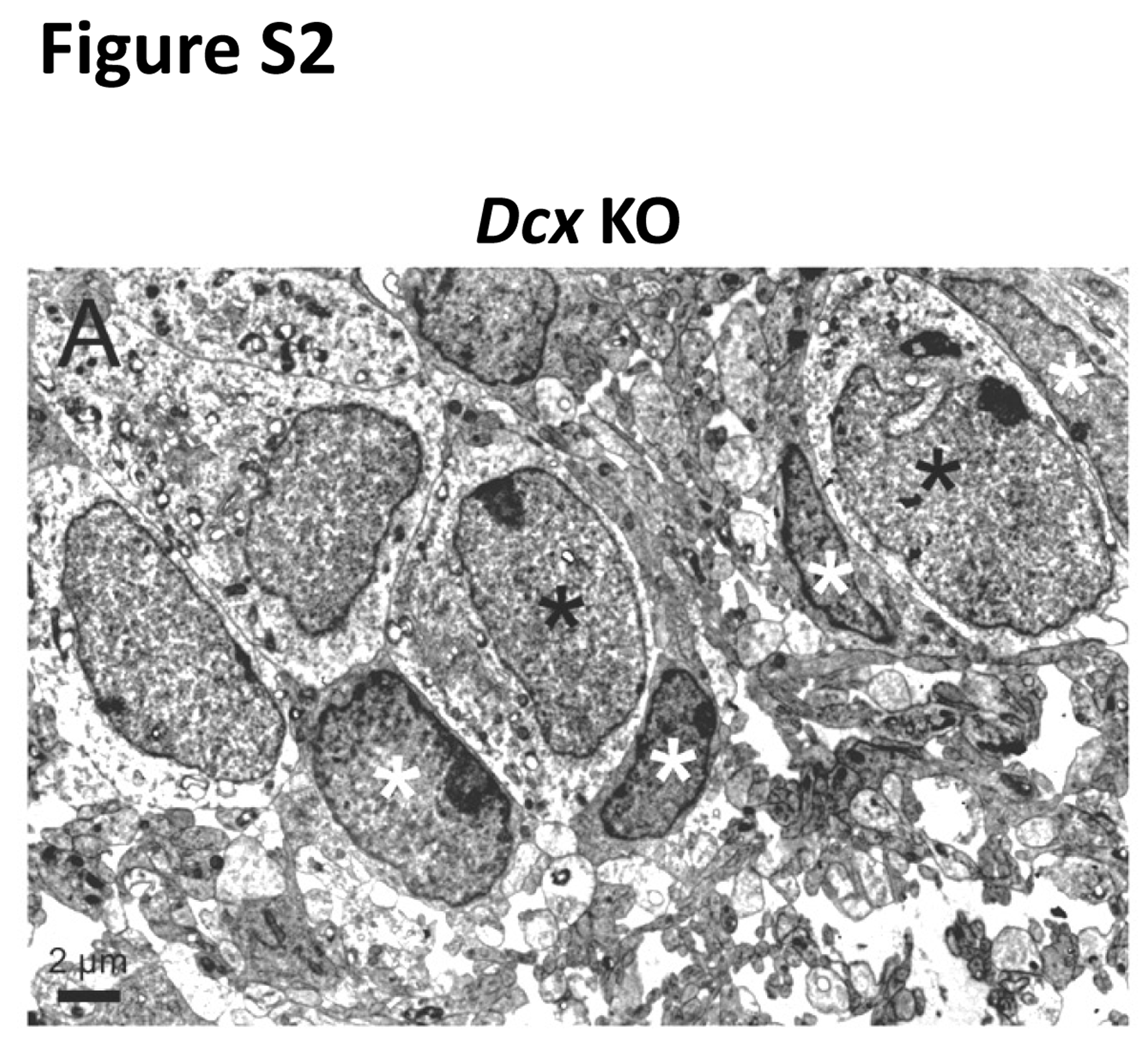

Supplement: Figure S2 — Arrangement of different cell types in the SPE pyramidal layer. (A) A region of the SPE layer of the Dcx KO hippocampal CA3 region showing neuronal-like cells (black asterisks) surrounded by other cell types with darker nuclei (white asterisks) and thin, dark cytoplasm. These latter cells appear to partially surround the neuronal-like cells. Scale bar: 2 µm. (TIF) [file pone.0072622.s002.tif]

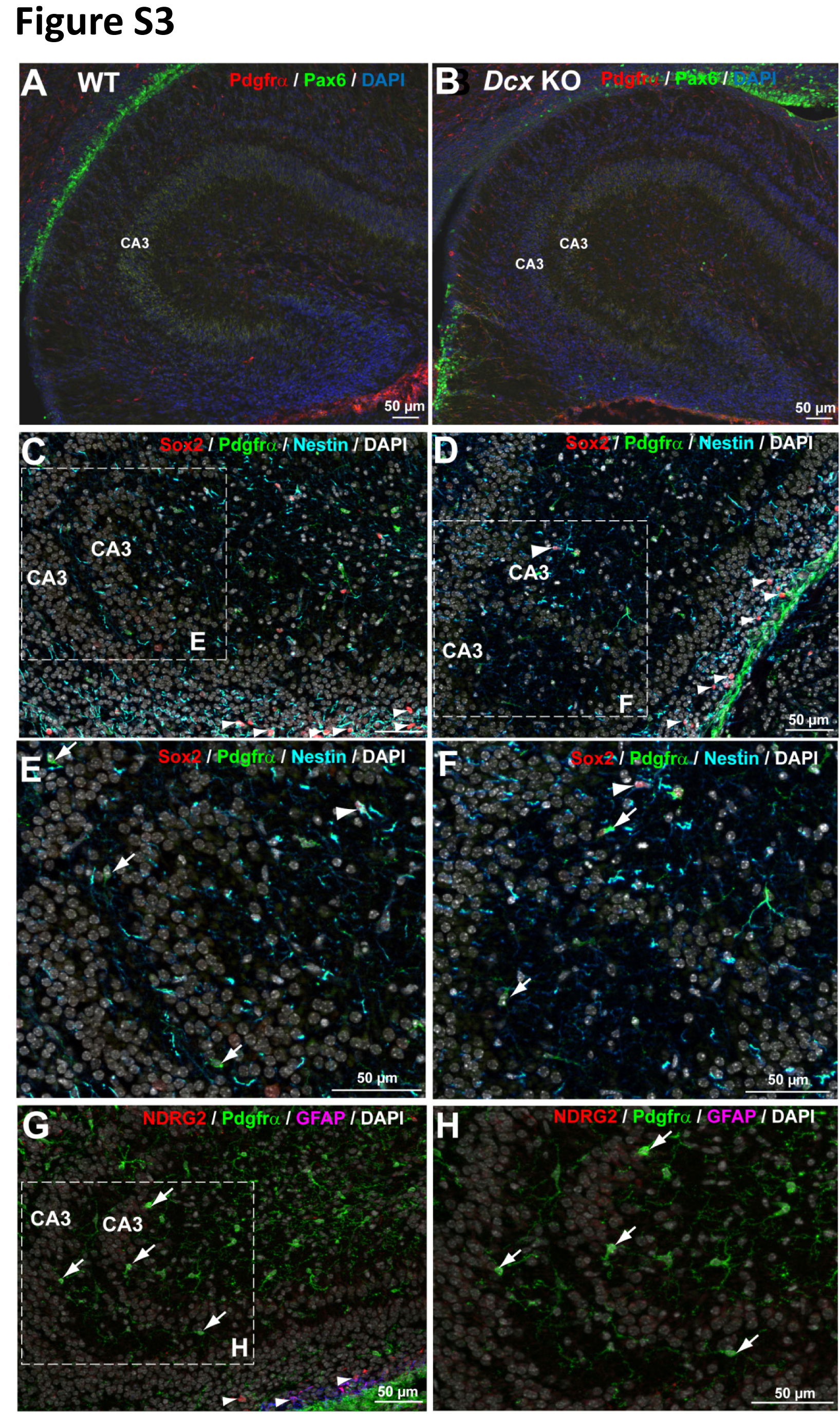

Supplement: Figure S3 — OPCs but not astrocytes intercalate between CA3 neurons. (A–H) Coronal sections of the CA3 region of the hippocampus of P0 WT (A) and Dcx KO mice (B–H). (A–B) Sections immunostained for Pdgfrα (red), Pax6 (green) showing that Pax6+ cells are only present in the ventricular region, but no ectopic Pax6 cells are found in the mutant CA3 SPE and SPI layers. (C–F) Sections immunostained with Sox2 (red), Pdgfrα (green) and Nestin (light blue) showing that no immature precursors are intercalated between CA3 neurons in Dcx mutant hippocampus. Note that some Sox2+ cells (arrowheads) can be found below the CA3 region as a stream of cells migrating towards the dentate gyrus (C) or in the stratum radiatum (E, F arrowheads). E and F are higher magnifications of boxed areas in C and D. (G, H) Sections immunostained with antibodies for astroglial markers NRDG2 (red), GFAP (pink), and OPC marker Pdgfrα (green) showing that only OPCs, but not astrocytes intercalate between the CA3 neurons in Dcx mutant hippocampus. Note astrocytes close to the ventricular surface (G, arrowheads). H is a higher magnification of the area in the inset of G. Scale bars: 50 µm. (TIF) [file pone.0072622.s003.tif]
